# Teacher Learning in Difficult Times: Examining Foreign Language Teachers’ Cognitions About Online Teaching to Tide Over COVID-19

**DOI:** 10.3389/fpsyg.2020.549653

**Published:** 2020-09-15

**Authors:** Lori Xingzhen Gao, Lawrence Jun Zhang

**Affiliations:** ^1^College of Foreign Languages, Taiyuan University of Technology, Taiyuan, China; ^2^Faculty of Education and Social Work, University of Auckland, Auckland, New Zealand

**Keywords:** COVID-19, language teacher cognition, online EFL teaching, information technology literacy (ITC), foreign language teaching and learning, China

## Abstract

The sudden global outbreak of COVID-19 in late 2019 has led to thriving online teaching, including the teaching of languages, across the world. As the online teaching of English-as-a-foreign-language (EFL) in Chinese universities is facing new challenges, EFL teachers have been positively exploring new solutions. To understand how EFL teachers were coping with the challenges, we set up this research as part of a larger study to examine EFL teachers’ cognitions about online teaching in response to the disruption of normal teaching plans. We did so by taking a qualitative approach through analyzing in-depth interviews with three EFL teachers from a Chinese university. Through thematic analysis we found that teachers had clear cognitions about features, advantages, and constraints of online EFL teaching and that they acquired information and communication technology (ICT) literacy through understanding students’ learning needs, online teaching practice, and the necessity of integrating traditional classroom teaching methods into online delivery. We conclude this study with a discussion on its pedagogical implications for similar contexts or colleagues facing similar challenges in other parts of the world.

## Introduction

The real-time coronavirus map updated in the early hours of March 28 on Google^1^ showed that the total number of confirmed cases worldwide reached 587,958, of which the United States topped the list with 104,011 cases and that European countries such as Italy (86,498), Spain (65,719), Germany (50,871), France (32,964), and the United Kingdom (14,590) etc. were severely afflicted. In Asia, China (81,394) and South Korea (9,332) ranked high in the disease-hit countries. Statistics of confirmed cases in the Middle East, Australasia, and Africa were not optimistic, either. Since late 2019, COVID-19 has swept over the whole globe as a worldwide concern and brought havoc to people’s health and life. Facing the high-risk challenge, to minimize the adverse effect of the epidemic, many countries have activated emergency response and implemented a wide range of measures, of which home-based quarantine and physical distancing are basic ones.

The epidemic situation of COVID-19 in China started in late December 2019 and worsened quickly in the first 3 months of 2020. The number of confirmed cases of COVID-19 increased rapidly. To control the sources of infection, cut off the channels of transmission, and make every possible effort to curb the spread of the disease, China’s central government enforced the policy of strict home-based quarantine and physical distancing among her people across towns and cities, while life-saving battles in hospitals and academic research into drug and vaccine development were going on at the same time. To protect the young generation from being affected by the coronavirus, the Ministry of Education of China called for online teaching and learning among teachers and students at all levels immediately afterward. Educational institutions were temporarily closed down. Online teaching therefore substituted the traditional way of classroom teaching and became the mainstream mode of delivering teaching.

For language teachers in Chinese universities, their teaching plans were disrupted and their knowledge and skills of ICT literacy were challenged. Due to the life-threatening global pandemic, language teachers, as well as teachers of other disciplines, had to move instruction online. In this process, the change in teachers’ cognitions about education and language teaching must have changed substantially. How to organize efficient activities via online teaching? How would students respond to online delivery, especially when the subject matter was about learning a foreign language? None of the teachers was sure about the effectiveness of such large-scale online language instruction. Worries and stress lingered on the teachers’ minds. How did EFL teachers in Chinese universities perceive and respond to their disrupted teaching when online teaching became the main mode of delivery? It is a question that needs urgent investigation as the teachers’ perceptions and responses to online teaching over COVID-19 greatly influence the quality of language education in Chinese universities. As part of a larger study, this paper presents a qualitative study, through in-depth interviews, on three EFL teachers’ cognitions about online teaching and their responses to, and strategies for, coping with their disrupted teaching. Unfortunately, little has been reported on how EFL teachers have responded to such a drastically challenging and evolving teaching and learning environment (cf. Fu and Zhou, 2020). This study was set up to fill the research gap. Our findings are expected to help language teaching professionals to understand how they themselves as frontline teachers can benefit from learning about how their colleagues were learning to cope with the new challenges by trying to understand online teaching platforms, online language teaching methods, online class management, among many other things, through scrutiny of three teachers’ cognitions and personal experiences.

## Literature Review

### Language Teacher Cognition

Research on teacher cognition began in the 1970s, thrived in the mid-1990s, and became a major area of enquiry in the field of foreign language education (Borg, 2006; Gao, 2019). The last 30 years has witnessed prolific research on language teacher cognition from a relatively new and undeveloped area into an important field of inquiry (Borg, 2019; Sun and Zhang, 2019; Li, 2020). The foci of language teacher cognition research include conceptual understandings of teacher cognition, factors affecting the development of teacher cognition, and the relationships between teachers’ cognition and their actual classroom instructional practice, among other things (Zhang and Rahimi, 2014; Gao, 2019).

According to Borg (2015, 2019), the concept of teacher cognition is complex, with about 60 distinctive terms used in language teacher cognition research to refer to it. Although the terms diverge in various contexts, the features can be classified into the following categories: Personal nature, mental lives, life or learning experiences, and the interactive influence of cognitive processes and instructional practice (Borg, 2003). Teacher cognition may involve teachers’ beliefs, knowledge, theories, attitudes, images, assumptions, metaphors, and conceptions about teaching, teachers, learning, students, subject matters, curricula, materials, instructional activities, among other things (Borg, 2003). In fact, teachers hold a wide range of practical theories which inform how they behave and teach in classrooms. Moreover, teachers’ practical teaching experiences contribute to the development of their cognitions (Kagan, 1992; Gao and Ma, 2011). The existing body of research shows that teacher cognition is defined, partly, by personal factors based on teachers’ own understanding of the practical classroom instructional activities (Clandinin and Connelly, 1987; Sun and Zhang, 2019). The body of research has a further, widely accepted implication: To better understand the process of teaching, both actions and cognitions underlying every decision that teachers make in their pedagogical practice need to be described and taken into consideration.

**FIGURE 1:**
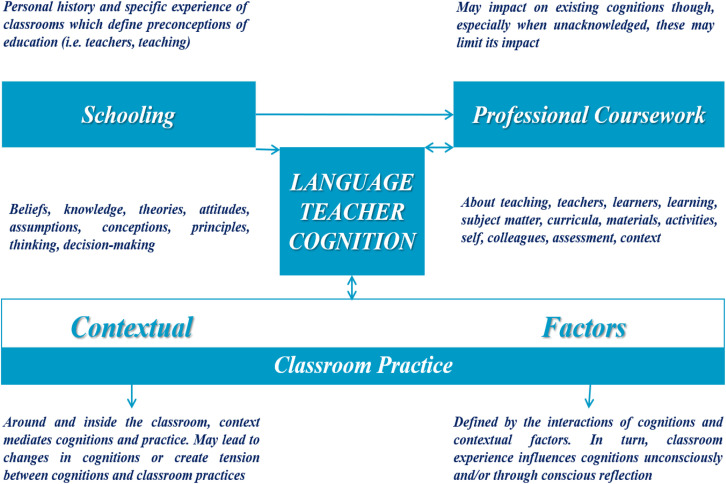
Elements and processes in language teacher cognition (adapted from Borg, 2015, p. 333).

The generally accepted conclusion about factors contributing to language teacher cognition is that it derives from teachers’ own learning experiences as language learners within formal classrooms (Pajares, 1992; Ellis, 2006; Nunan and Richards, 2015), and through “apprenticeship of observation” (Borg, 2003) in early teaching experiences and teacher training courses (Popko, 2005), which significantly influence the way in which they view and approach teaching (Sun, 2017; Sun and Zhang, 2019). As Richards and Lockhart (1996, p. 30) claimed, other sources may include “teachers’ personality factors, educational principles and research-based evidence,” different stages the teachers are in Berliner (1994), teachers’ emotions (Zembylas, 2005), and language policy (Farrell and Kun, 2007). In short, many sources contribute to the formation of teacher cognition. Borg’s (2015) model intends to capture the potential factors affecting language teacher cognition, which is presented below (see Figure 1).

As shown in Figure 1, the three categories that influence teacher cognition are schooling, professional coursework, and contextual factors (classroom practice). For schooling, teachers’ personal history and specific experience in classrooms help define their early cognitions and shape their understanding of teachers and teaching. Teachers’ professional coursework also may have an effect on their existing cognitions, although its impact may be limited because teachers are unaware of the relationship of their coursework to their practice. Contextual factors are related to, and, mediate cognition-practice relationship inside the classroom context and can cause teachers’ cognitions to change or form a tension between their cognitions and instructional practice. Classroom practice is bounded by the interactions of cognitions and contextual factors, and in turn, teachers’ classroom practice influences their cognitions in an unconscious and/or conscious way.

How teachers’ cognitions are related to their actual classroom instructional practice is increasingly a research focus in the field of teacher education, including the cognition-practice congruence, that is, the extent to which teachers’ instructional practice is consistent with their cognitions and any inconsistency between the two (Borg, 2019; Sun and Zhang, 2019). Teachers possess theoretical beliefs about teaching, and their beliefs and cognitions provide a basis for their teaching behavior (Borg, 2011; Basturkmen, 2012; Zhang and Ben Said, 2014; Farrell and Ives, 2015; Kaymakamoglu, 2018), and their beliefs guide their thought and behavior. Inconsistency between teachers’ stated cognitions and their observed classroom practice has been reported in case studies of experienced and novice language teachers. Such studies were carried out with teachers in primary schools (Farrell and Lim, 2005), secondary schools (Ng and Farrell, 2003), high schools (Mitchell, 2005), and tertiary settings (Sun, 2017), and they have examined topics ranging from beliefs about, for example, teachers’ roles (Anstrom, 2003), communicative language teaching (Sugiyama, 2003), teaching methods (Tucker, 2001), and students’ needs (Gilliland, 2015). The inconsistency between teacher cognition and practice is also identified in the research in a number of areas (Richards, 1998) in mainstream education (Fang, 1996), literacy education (Cummins et al., 2004), second/foreign language teaching (Xiang and Borg, 2014; Sun, 2017), and the teaching of specific skills in English as a foreign language, including grammar (Graus and Coppen, 2016), writing (Zhang, 2016a,b), reading (Vaish, 2012), vocabulary (Gerami and Noordin, 2013), corrective feedback on oral communication (Zhang and Rahimi, 2014; Rahimi and Zhang, 2015), speaking (Baleghizadeh and Nasrollahi Shahri, 2014), pronunciation (Buss, 2016), and corrective feedback on pronunciation (Cooper, 2019). The degree of inconsistency, from partial congruence to clear divergence, with a low positive relationship or limited correspondence, is also reported in the research.

### Language Teachers’ ICT Literacy

The benefit of technology for language teaching and learning has been highlighted in many studies; its role is not only to engage learners in the learning process, but also to promote learners’ motivation and learner-centered instruction (Chapelle, 2005). The benefit has been further enhanced and expanded by virtue of technological advancements which have introduced mobile devices such as smart-phones and tablet computers to language learning contexts. Teaching and learning of languages have been enabled to transcend time and space limitations and made “more fun and interactive” (Demouy et al., 2016, p. 19). The convenience, mobility, and effectiveness of mobile learning have become evident (e.g., Evans, 2008; Liu et al., 2008). The benefits of mobile learning should be extendable to EFL contexts as well. The integration of mobile technology into EFL teaching and learning is thus feasible in ways that facilitate students’ enhancing their language competence. Although mobile learning seems to be more central to learners than teachers, guidance and advice from teachers are always required by students (Kukulska-Hulme, 2009). However, teaching with technology can be a complicated and difficult task for some teachers under the influence of social and contextual factors (Koehler et al., 2011). Therefore, before applying mobile learning, teachers need to learn and grasp adequate technological and pedagogical knowledge (Tai et al., 2015). The technological pedagogical content knowledge (TPACK) model, as adopted in our study is a framework that can help us understand how teachers think and take actions accordingly.

**FIGURE 2:**
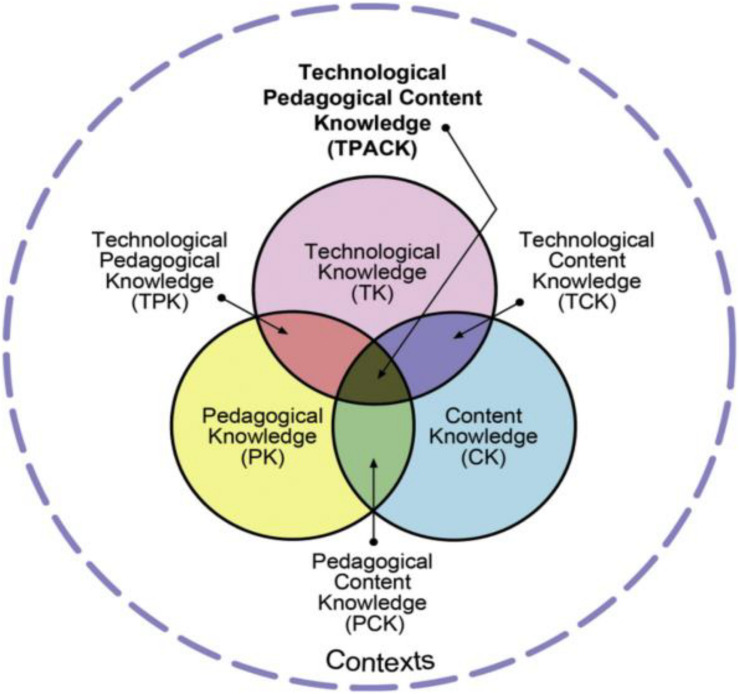
TPACK model (Source: tpack.org).

Based on the original work of Shulman (1986), where he proposed his conceptualization of teacher knowledge through his pedagogical content knowledge (PCK) model, the TPACK model was developed by Koehler and Mishra (2005) as a way to describe the relationships and interactions between teachers’ knowledge of technology, pedagogy, and the subject matter, as shown in Figure 2.

As is clear in Figure 2, teachers’ technological knowledge (TK, i.e., knowledge of how technologies should be used in the content domain), pedagogical knowledge (PK, i.e., knowledge of how contents are learned and taught), and content knowledge (CK, i.e., knowledge of the subject matter to be taught and learned) are connected to one another and lead to three new knowledge forms: PCK, technological content knowledge (TCK), and technological pedagogical knowledge (TPK). The interrelationships of these three forms of knowledge give rise to TPACK, the core concept central to the TPACK model. TPACK, as the term denotes, is characterized by the integration of technological knowledge and pedagogical knowledge into the teaching and learning of content knowledge. While the concept of TPACK gathered the attention of many education researchers, any elaboration on its relevance to specific subject domains such as language teaching and learning remains limited. Chinese university EFL teachers’ information and communication technology literacy, as a form of TPACK, in online EFL teaching during COVID-19 has been scarcely explored in the field of second or foreign language education.

### Online Teaching Over COVID-19 in China

Teachers’ ICT literacy is not a new topic; instead it is an important component in the Chinese Ministry of Education’s effort for modernizing education in China, as emphasized several times in various official documents. In 2016, the Ministry of Education of China issued *The 13th Five-Year Plan of Education Informationalization*, which called for the integration and development of technology and education, creation of a good environment of informationalized education, and upgrading of teaching concepts, modes and content to train talents for the information age. *The Guideline for College English Teaching* (Ministry of Education of China, 2017) pointed out that as teachers’ quality and competence are key factors affecting teaching quality, university English teachers must actively upgrade their knowledge and skills in modern education technology and adapt themselves to meet the needs of university English teaching in the technology-enhanced learning and teaching environment. In the *China Education Modernization 2035*, it is proposed that education reform be carried out through the cultivation of a high-quality, professional and creative teaching contingent to quicken the mode of talent training with modern technology in the information age.

The abrupt outbreak of COVID-19 in China in late December 2019 severely disrupted people’s regular schedule of life, education and work. Universities were not exempted at all. Lessons at all levels scheduled for the Spring Semester 2020 were being delivered online instead of face-to-face in the classroom. Shouldering a historical mission of education amid the COVID-19 crisis, scholars and teachers have researched online teaching recently in the field of education in recent weeks in other parts of the world. However, due to the scarcity of time and lack of facility to carry out empirical research, our literature search resulted in only three publications on Chinese EFL teachers related to online teaching over COVID-19 and in the section below we review them for the purpose of helping us to understand the challenges EFL teachers faced and their coping strategies in having to deliver online teaching.

The first paper reports a study by Fu and Zhou (2020) that investigated the opportunities and challenges of online teaching. The authors reported that the hardware facilities and wi-fi conditions are uneven across schools and areas; teachers’ information technology skills and resilience cannot meet the needs of online teaching; the online resources and platforms are insufficient for online teaching; students, parents, and schools have different expectations of online teaching; the need for quality individualized education cannot be satisfied by online teaching. In view of these challenges of online teaching, the authors suggested optimizing wi-fi and hardware facilities and integrating quality online resources and platforms be seriously considered.

The second publication by Jiang et al. (2020) discussed the principles and mode of online teaching over COVID-19. The principles of online teaching over COVID-19 the authors reported include simplicity in technology, immediate feedback, interactive communication, and precision management. Their suggestions are consistent with the countermeasures and suggestions for enhancing the quality of online teaching proposed by Wang et al. (2020), which is the third paper we review below.

Wang et al. (2020) explored the advantages and rapid development of online teaching in China during COVID-19 and the benefit of the assistance from 5G technology in improving teaching and learning. They proposed a range of suggestions for improving online teaching quality and efficiency. The suggestions include creating a smart environment for home-based teaching and learning, choosing suitable online teaching modes, and enhancing interaction in online teaching. These are all useful suggestions, but unfortunately, they are not based on empirical evidence or supported by any empirical data.

The literature review shows that though language teacher cognition and teachers’ information technology literacy have been profoundly and systematically researched in terms of conceptual understanding, relationships among components within each model, and application on various education levels and in various subject areas, no exploration has been carried out into language teacher cognition about online EFL teaching in China, especially language teachers’ cognition about their information technology literary for online EFL teaching over COVID-19. The available research into online teaching over COVID-19, in spite of its paucity, provides insights into further research. Our study is an attempt to investigate three language teachers’ cognitions about online EFL teaching and their information technology literacy in response to disrupted teaching plans in a Chinese university. To be more exact, the study addresses the following research questions:

(1) What are the language teachers’ cognitions about online teaching over COVID-19?
(2) How did teachers acquire ICT literary in the initial stage of COVID-19?

## Materials and Methods

As part of a larger study, the study reported here is a qualitative inquiry into what the language teachers think about, how they carry out and reflect on online EFL teaching over COVID-19, trying to construct meaning from their experience, feelings, and thoughts in this trying time. We carried out this study by framing it within the theory of constructivism. According to constructivism, reality is constructed by individuals interrelating with their cultural and social world: Human beings, and by necessity, including teachers, seek understanding and perceptions of the real world where they live and work, and develop subjective understanding from their own experience. As Merriam (1998) advises, qualitative researchers are interested in understanding the meanings people construct, and this was the purpose of our study as well.

### Participants

#### Sampling Strategy

The participating teachers in the present research were chosen through convenience sampling due to the sudden outbreak of COVID-19 and teachers’ subsequent festinate preparation of online EFL teaching. Convenience sampling is “selecting a sample based on time, money, location, availability of sites or respondents, and so on” (Merriam and Tisdell, 2016, p. 98). Through convenience sampling we contacted some EFL teachers from a university in a Northern Chinese city. They expressed their concerns, worries, and anxieties over the influence of epidemic on EFL teaching and their follow-up online instruction. After initial talks, three participants agreed to take part in our study. Their backgrounds are described in the following section.

#### Participant Backgrounds

The participants in this research are three teachers of English from a university in a Northern Chinese city. They were chosen mainly because the courses they taught online represented three different types: Comprehensive English, English Listening, and Linguistic Pragmatics. At the same time, the three teachers’ educational backgrounds and teaching experiences varied substantially. To protect personal information and for the convenience of data presentation, the participants are referred to as Jane, Mary, and Shirley. Table 1 provides an overview of these teachers’ demographic information:

**TABLE 1:** Overview of participants’ demographics.

**Name**	**Gender**	**Age**	**Educational degree**	**Years of teaching**	**Course**	**Students’ university year level**	**Online platforms**
Jane	Female	39	Master of Arts	18	Comprehensive English (IV)	2	Chaoxing, QQ
Mary	Female	49	Ph.D. in Arts	27	English Listening (I)	1	Ding Talk
Shirley	Female	28	Ph.D. in Arts	1	Linguistic Pragmatics	3	Chaoxing, QQ, Baidu Netdisk

As shown in Table 1, Jane, Mary and Shirley bear differences in their ages, educational backgrounds, years of teaching, courses taught, students’ levels, and the online platforms they used. In terms of students’ level, the numbers in Table 1 represent their grades; namely, students in level 1 refer to freshmen, whose English proficiency is lower than level 2 (sophomores) and 3 (juniors). These differences among the participants present a diverse coverage of online EFL teaching. Their online teaching had been conducted for 6 weeks when the data were collected, during which their online teaching underwent a process of design, trial, and stabilization. The three participants are typical of EFL teachers in the university.

### Interviewing as a Research Tool

Methodologically interviews are used frequently in educational research because they are an instrument that can be used for in-depth investigation into the issue at hand (Miles et al., 2014). According to Creswell (2014), interviews can be carried in a face-to-face and one-on-one way, by telephone, in focus groups, or through the Internet (emails, for example). Interviews involve generally open-ended questions, which are few in number and designed to elicit views and opinions from the participants. Interviews are a powerful data collection instrument for qualitative research as they are useful when participants cannot be directly observed; through interviews participants can provide historical information; interviews allow the researcher to control over the line of questioning. Interviews are thus a natural and socially acceptable way of collecting qualitative data in various situations while focusing on diverse topics for in-depth information (e.g., Zhang, 2010). The interviewer can adopt flexible approaches and probe into newly emerging issues, and the interview protocol helps to keep the interaction on the right track, which is a systematic coverage of the domain (Rubin and Rubin, 2011). However, the disadvantages of interviews cannot be denied and neglected. Interviews provide indirect information filtered through the views of interviewees; interviews provide information in a designated place rather than a natural setting; the researcher’s presence may bias responses; not all people are equally articulate and perceptive.

According to the degree of structure, the one-to-one interviews can be divided into structured interviews, unstructured interviews, and semi-structured interviews. Structured interviews and unstructured interviews are two extreme types of interviews where the interviewer either closely follows a prepared and elaborate interview schedule with little room for variation in the responses or follows the interviewee in unpredictable directions with interruptions kept to a minimum. Semi-structured interviews offer a compromise between the two extremes; with a set of pre-prepared guiding questions and prompts, the interviewer provides direction and guidance and at the same time allows the interviewee to elaborate on some issues (Dörnyei, 2007).

Interviews are suitable and appropriate for the present research as its intention is to gather information about the teachers’ cognitions about online EFL teaching and their acquisition of ICT literacy in the initial stage of online EFL teaching. The semi-structured interview was adopted as the main tool to elicit verbal data for this study. The first author worked as the interviewer and asked two broad open-ended questions to direct the interview, and the participant/interviewees were encouraged to give detailed responses to the following two broad questions: (1) What do you think of online EFL teaching over COVID-19? (2) How did you acquire your ICT literacy in the initial stage of COVID-19? The semi-structured interview was used in the present research because it avoided the disadvantages of both structured interviews and unstructured interviews.

### Data Collection Procedures

The data for the present study were collected from March 25 to 28 through WeChat interviews with Jane on a one-to-one basis. Jane was interviewed three times and each interview lasted 45 min or so. Mary’s and Shirley’s data were in the form of written summaries and reflections on their online EFL teaching based on their responses to the two interview questions. Table 2 below is a record of the interviews with the three participants.

**TABLE 2:** Statistical record of data collection.

**Name**	**Time**	**Date**	**Duration**	**Mode**	**Main topic**	**Form of data**
Jane	1	March 25	48 min	WeChat	Cognitions about online EFL teaching	Texts, audio clips
	2	March 26	47 min	WeChat	Preparations for online EFL teaching	Texts, pictures, video clips
	3	March 28	48 min	WeChat	Reflections on online EFL teaching	Texts, audio clips
Mary	Written summaries and reflections on online EFL teaching					Texts, pictures, screenshots, etc.
Shirley	Written summaries and reflections on online EFL teaching					Texts, pictures, screenshots, etc.

As Table 2 shows, Jane was interviewed three times via WeChat. In the first interview, Jane shared her cognitions about online EFL teaching. The data were in the form of texts and audio clips. In the second interview, Jane introduced her preparations for online EFL teaching and showed the pictures/screenshots and video clips she made prior to her online teaching. In the third interview, Jane updated the interviewer with her reflections on online EFL teaching with data in the form of texts and audio clips, etc. Mary’s and Shirley’s summaries and reflections on online EFL teaching were mainly texts, pictures, and screenshots. The collected data include texts, pictures/screenshots, audio clips, and video clips, which helped to ensure the trustworthiness of the present qualitative study. Besides the interview data, the author took field notes as well during and after the interviews. Having different forms of data also made possible the triangulation of data. The data collection ceased when there were sufficient data for the exploration of the participants’ cognitions about online teaching and their teaching practice during the period of COVID-19.

### Data Analysis

The data analysis procedures began immediately after the first interview by both authors. Subsequent data in audio clips collected from the interviews were transcribed and processed. In addition, the notes taken during each interview were typed, read, and summarized. They were then processed through thematic analysis. Thematic analysis is a method for identifying, analyzing and reporting patterns (themes) emerging from data (Braun and Clarke, 2006; Miles et al., 2014), during which process the researcher scrutinizes the data for typical themes and concepts (Miles and Huberman, 1994; Rubin and Rubin, 2011).

During the interview transcriptions, no translation was done of the non-English data because the original language from the participant could better convey their meanings in their first language, mandarin Chinese, which is also the first language of both authors, which means that they are fully understood for meaningful analysis. This decision was made because as stated by Wang et al. (2020), “the relation between subjective experience and language is a two-way process; language is used to express meaning, but the other way round, language influences how meaning is constructed” (pp. 313–314). Much information will be lost in the course of translation because of the lack of equivalent vocabulary, syntax, idioms, and concepts between the source language and the target language (Sechrest et al., 1972). In doing the analysis we were constantly reminded of these possible shortcomings and therefore we mainly followed the original Chinese interviews or texts. For the purposes of reporting the findings that can be understood by a larger international audience, all the interviews were presented in English.

**FIGURE 3:**
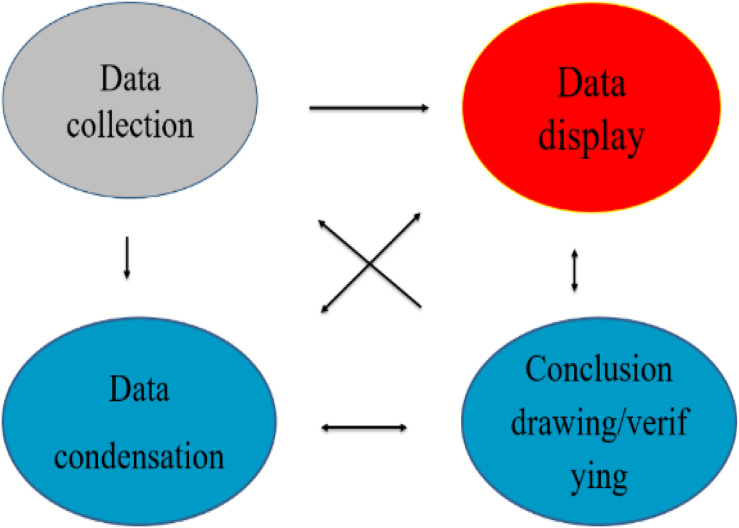
Components of data analysis: Interactive model (adapted from Miles and Huberman, 1994; Miles et al., 2014).

Our data analysis was also guided by the Interactive Model (see Figure 3). As is shown, the condensation, display, and conclusion drawing/verifying are closely interwoven or connected to data collection, despite the whole process being called “analysis” in the general sense. Inspired by the Interactive Model, we created a five-step data analysis procedure (see Table 3).

**TABLE 3:** Five-step data analysis model used in the present research.

**Steps**	**Description of the process**
1 Cleaning the original data	Reading through the original data, find and correct the errors in the original data files, check the consistency of the data, etc.
2 Coding the data	Repeatedly reading the original data and generating open codes.
3 Generating themes	Comparing the original codes, checking the relevance between codes, and naming the themes.
4 Categorizing the themes	Putting the themes into different categories according to certain standards and principles.
5 Producing the report	Producing a scholarly report of the analyzed data with vivid, compelling extract examples, relating back to the research question and literature.

## Findings and Discussion

### Teachers’ Cognitions About Online EFL Teaching Over COVID-19

The sudden outbreak of COVID-19 across the whole globe posed a crisis to traditional face-to-face ways of EFL teaching in Chinese universities. In the meantime, it also provided an opportunity for all parties involved to update their cognitions about EFL teaching and upgrade their knowledge and skills of information technology literacy to meet the needs of online teaching. We cannot reset 2020, but it can be argued that we can reset ourselves with updated cognitions and upgraded knowledge and skills of information technology literacy.

#### Teachers’ Perceptions of Online EFL Teaching Over COVID-19

The participating teachers had diverse perceptions of online EFL teaching over COVID-19 as they compared it with traditional classroom language teaching to explore the features of online EFL teaching. Based on their online teaching experiences, they looked at online EFL teaching from different perspectives. Jane expressed her view of teaching and learning as limited by teachers’ mastery of information technology in online EFL teaching:

Jane: *Online teaching is diametrically different from traditional classroom teaching. In the traditional teaching mode, teachers prepare the lessons and deliver them in the classroom, and interact with students face-to-face, relying little on information technology and the network infrastructure. They can fulfill their teaching tasks within the specified time. However, online teaching is conducted in a virtual space, in which face-to-face interaction can hardly be achieved with the same effect as in a physical classroom. Limited by the online teaching conditions, it is difficult to guarantee the full participation of students; the learning outcomes are closely related to self-management and metacognitive ability on the students’ part.*

As reflected in Jane’s observation, online EFL teaching was totally different from traditional classroom teaching because of its heavy reliance on information and communication technology, which subsequently leads to uncertain learning outcomes on the students’ part. Jane’s negative view of online EFL teaching was a result of her worry and anxiety about the new and unfamiliar form of teaching in the initial stage. If a chronological case study can be conducted with Jane, findings concerning the change in her ideas of online EFL teaching will be expected. Different form Jane’s view, online EFL teaching is “new and efficient extension of traditional classroom teaching” in Mary’s and Shirley’s eyes. Mary in details illustrated her favorite idea about online EFL teaching in comparison with traditional classroom teaching:

Mary: *Online EFL teaching expands traditional classroom teaching in three main aspects. First, such online learning platforms as MOOC provide quality resources for teaching and learning, from which the teacher can choose some key content for the students. Second, in terms of supervision and assessment of students’ learning process and outcome, more objective records can be retrieved from online teaching. Third, the teacher–student interaction in online teaching can take more forms. Students are more active because they don’t need to show their image as they did in traditional classroom teaching which effectively emboldens their courage and confidence in answering questions. Furthermore, the students’ adoption of various forms of participation (such as words, pictures, animations, Emojis, and voice messages, etc.) in online teaching livens the atmosphere.*

Online EFL teaching, in Mary’s eye, is more positive than negative. Mary’s perception about the advantages of online EFL teaching was echoed and supplemented in Shirley’s written reflection:

Shirley: *Online teaching of EFL courses has its unique merits. Students can repeatedly watch teaching videos to meet their learning needs. Teachers can keep track of students’ progress and provide targeted support and advice.*

In spite of their optimistic views about online EFL teaching, Mary and Shirley didn’t ignore its drawbacks. Mary explained her structured understanding of the disadvantages of online EFL teaching related to teacher–student interaction, reflection of students’ learning progress, and inequality in education caused by imbalance among network devices across regions.

Mary: *Online EFL teaching has its insuperable weaknesses in terms of timely teacher–student interaction, reflection of students’ learning development, and requirement of network devices on students’ part. First, none of the online teaching platforms can guarantee instantaneous interaction between teachers and students, which subsequently brings negative effect on the teaching efficiency. Due to the time lost in the lengthening of teacher–student interaction, online teaching capacity is less than that of traditional classroom teaching and a lot of work needs to be assigned for the students to do after class. Second, as the teacher cannot see every student in online teaching, it is more difficult to monitor their learning, not to mention their after-class assignments. Third, the imbalance of network devices across regions inevitably leads to inequality in education among students.*

Mary’s practice-driven insight into online EFL teaching reflects her thorough understanding of teaching and learning process of EFL courses, which is in turn central to her online teaching practice. Several weeks of online EFL teaching experience widens her understanding of language education and enriches her cognition of online teaching in particular. Mary’s thought about the drawbacks of online EFL teaching was overall and general, which was fortunately remedied by Shirley’s reflection based on her online classes.

Shirley: *In my online classes, two things are found to hinder teaching efficiency. Prepared teaching materials turn out to be too much for the online class due to my inexperience and unpredictable network signal. Online class activities such as open discussion are inappropriate.*

In addition to the features of online EFL teaching, the change in the role of teachers is also one of the major themes emerging from the data. Jane stated that the role of the teacher changed from the traditional knowledge imparter and the classroom activity organizer to the resource integrator and the supervisor for students’ autonomous learning in online teaching mode. She also mentioned that in her online teaching practice she carefully chose online resources provided by leading publishers in foreign language teaching, such as Foreign Language Teaching Press (Beijing) and Foreign Language Education Press (Shanghai), as effective supplementary materials to the textbooks adopted for the coursed she taught.

#### Challenges Facing EFL Teachers Teaching Online Over COVID-19

Jane, Mary, and Shirley all expressed their confusion and anxiety during the initial stage of online teaching. In her interview, Jane talked about her psychological pressure during her preparation for online EFL teaching, and mentioned that her worries were mainly about her lack of proper information technology literacy for online teaching, the insufficient conditions for online teaching and learning on both teachers’ and students’ part, and invalid class management during online teaching.

Jane: *What platform should I use for online teaching? Will it collapse during online teaching? What if unexpected breakdown happens and I am not able to solve it? I am so ill-prepared and worried.*

The first challenge Jane met is typical of EFL teachers and central to online teaching. Teachers were familiar with teaching methods in face-to-face delivery in classrooms before the abrupt breakout of COVID-19 and their information technology literacy was limited to the integration of digital equipment into classroom teaching, with little knowledge and skills for online teaching. This put EFL teachers in a poor position, which restricts them from conducting online teaching effectively especially when they are supposed to do so after a very short period of training. Their uncertainty over the platform, channel, and specific skills, etc. for online teaching poses a challenge before them.

The second challenge is insufficient network conditions for online teaching and learning. Online teaching and learning requires a large amount of data transmission and thus relies heavily on the wi-fi infrastructure or monthly data plans. This is costly for students, too. If a flaw occurs in one segment, the whole online class will be forced to stop. And Jane also worried about the materials for online teaching and learning. As COVID-19 broke out in late December 2019, the students were having winter vacation with their family. After she contacted the class representative, usually called “class monitor” in China, she found that few of the students took their textbooks along when they left the university. She was concerned that such situations might hinder her successful online teaching and learning.

The third challenge Jane met was the possible invalid class management during online teaching. Since the teacher and students were not in one actual room during online teaching, as is usually the case, class management became more challenging. The teacher were not able to observe the students and give timely feedback through non-verbal means such as eye contact. The inefficiency in class management may lead to students’ idling away and unsatisfactory learning outcome.

#### EFL Teacher’s Readiness to Deliver Online Teaching to Tide Over COVID-19

In view of the three challenges, Jane, Mary, and Shirley did a lot of autonomous learning and exploration of relevant elements and technological skills. As a result, they found the solutions and became ready for the online teaching.

To solve the first challenge, they learned how to use various Internet platforms such as Chaoxing, Ding Talk, MOOC, etc. for online teaching, and some chat groups in social networking apps such as WeChat and QQ. These platforms and apps have been developing fast since the outbreak of COVID-19. Through learning and trials, they got familiar with their functions including live streaming, uploading, and downloading files, and roll calling, etc. They were also aware of the advantages and disadvantages of each platform and app. The ability to make a choice of suitable online platform(s) prepared them for online EFL teaching and enhanced their confidence in information technology for online teaching.

Facing the second challenge, the three participants all contacted the students for accurate information and took corresponding measures. In Jane’s case, she contacted the class monitors 2 weeks before online teaching began and updated her knowledge about the situation of her students and their readiness for online learning. What the monitors told her made her come to a comfortable realization that her preparedness for online teaching met her students’ expectations.

Jane: *The monitor told me that most of the students in her class left their textbooks (Contemporary College English Book Four) in their dormitory when they left for home. Instead they took Exercise books for TEM-4 with them and planned finish the exercises in winter vacation. As for Internet access, every student has wi-fi at home and smart devices for online learning.*

After she knew the students’ lack of textbook, Jane asked the monitors to search for the PDF version of the textbook. One day later, this was solved and every student in the class was equipped with the basic learning material – the textbook.

As for class management, Jane sorted the students into learning groups of four or five, assigned learning tasks related to the textbook or the *Test for English Majors – Band 4 (TEM-4)* grammar and vocabulary for each group and shared their learning outcome in the online platform. This worked well in the past 6 weeks, as it kept every student engaged in learning activities. The sharing and feedback encouraged the students to continuously perform for better.

### Teacher’s Acquisition of ICT Literacy in the Initial Stage of COVID-19

Three themes related to teachers’ acquisition of ICT literacy emerged. In Jane’s case, her ICT literacy was acquired as a result of a clear understanding of her students’ learning needs; it was also acquired through online teaching practice; her integration of traditional classroom teaching into online teaching with information technology increased her confidence. These themes were also embodied in Mary’s and Shirley’s cases.

#### ICT Literacy Acquired Through Clear Understanding of Students’ Learning Needs

In Jane’s case, before starting the online teaching, Jane was fully aware of her students’ learning needs in the Spring Semester. She mentioned her cognition when she talked about her preparations for online teaching.

Jane: *My main teaching task this semester is Comprehensive English (IV) for English 1801/1802. The second semester of the sophomore year is one of the most critical semesters for English majors in universities. Along with completing the heavy learning tasks of various courses, they are also required to take part in Test for English Majors Band Four (TEM-4) which is supposed to check the learning outcome in their first 2 years of university.*

As is the rule in Chinese universities, English majors’ performance in TEM-4 is very important for the students, failing which they will be deprived of their degree. In the arrangement of tutoring students to prepare for the exam, the course *Comprehensive English* is customarily responsible for two blocks: *Grammar and Vocabulary*, and *Cloze.* These two blocks in TEM-4 are difficult for the students but take a high percentage in the total marks. Students’ learning needs put forward higher requirements and challenges, and therefore bring great psychological pressure to Jane and the students. With a clear understanding of the students’ learning needs, Jane made a neat choice of teaching materials and teaching methods as well as the most suitable platform for online teaching: Chaoxing. Jane’s choice of Chaoxing Platform for online teaching was a result of guidance from her university and college.

Jane: *Under the guidance and help of the university and college, I quickly got familiar with the operation process of Chaoxing Platform for online learning, and decided to choose it as the main platform for my online teaching platform.*

The Chaoxing online platform has multiple functions and evident advantages as it is specially designed for online teaching and learning: Required textbook chapters and other editable teaching materials make the platform user-friendly, integrated, and systematic; Chaoxing’s cloud drive has a large storage capacity, and the data stored in its cloud drive can be synchronized to its mobile phone app for convenient operation; rich online classroom activities in Chaoxing, such as roll calling, voting, topic discussion, question posing, surveys, etc., basically cover activities in offline physical classroom teaching, and effectively promote classroom activities and improve online teaching efficiency; the notification function is very practical in that it cannot only quickly send notifications to targeted students, but also report the number of students who have read the notification in time to the teacher and be operated to remind those who have not read of reading in time; the homework function provides set an array of question forms, such as multiple choice, gap filling, short answer tasks, etc., in line with the actual needs of teaching, and this function can also grade some homework, which effectively reduces the workload of teachers. The multiple functions of Chaoxing help Jane get familiar with online teaching process, master online teaching methods and skills as soon as possible, and ensure the teaching efficiency of her online teaching.

Shirley’s case was similar to Jane’s. She chose Chaoxing as her main platform for online teaching and QQ chatgroups for class discussion and Baidu Netdisk for transmitting learning materials.

However, in Mary’s case, as she recognized in the *English Listening* course that students needed to do a substantial amount of practice to acquire the language skill, she chose Ding Talk as the online teaching platform for live teaching of the course. Such platforms as MOOC and Chaoxing were conceived by her as improper platforms for online learning of *English Listening* because “they emphasize knowledge transference instead of student-centered practice.” In her Ding Talk live class, she directly engaged the students in the listening practice like in a traditional classroom. She started a lesson with providing the students with knowledge about new words and cultural background of the listening item. Then, she played the audio clips four times to the students and asked them to do follow-up exercises during the intervals and provided them with necessary support such as the answers to the exercises and the audio scripts. She asked the students to upload their notes of listening to Ding Talk for a further look at their mistakes and gave them corresponding guidance.

#### ICT Literacy Acquired Through Online Teaching Practice

**FIGURE 4:**
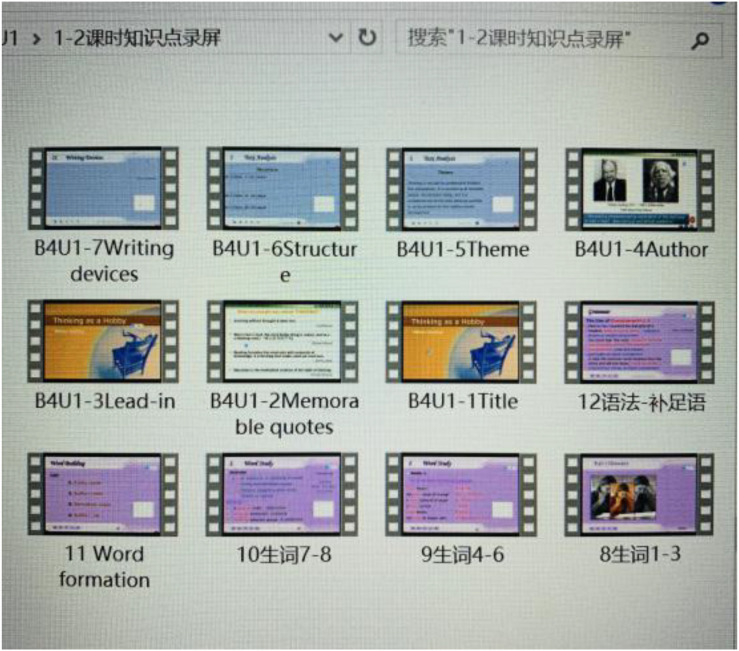
The “PPT + Voice” files made by Jane.

Jane encountered many difficulties and challenges in preparing for her online teaching, such as making files containing both PowerPoint slides and voicing over her PowerPoint slides (PPT + voice), uploading teaching materials to the Chaoxing cloud drive. The first challenge Jane encountered during her preparation for her online teaching was recording the files with EV Capture. Figure 4 below shows the “PPT + voice” files made by Jane.

Jane: *When I made the first “PPT* + *voice” files, I was unfamiliar with the software and often made operational mistakes. In the meantime, without students’ response as in the traditional classroom, I was always unsatisfied with the sound quality and atmosphere of recording: it was not lively as supposed to be. Then I need to repeatedly abandon the unfinished files and rework on them from the very outset. As a result, a presentation of a few minutes can take an hour, which upsets me at the end of the day.*

The difficulty in video recording had Jane stumped for quite some time, and then she solved the problem by adding classroom teaching skills such as raising questions after knowledge explanation, waiting some seconds for students to digest, and providing feedback to imaginary answers from students.

Jane: *With imagined teacher–student interaction added, the “PPT* + *voice” files has become more vivid and lively.*

The second challenge Jane encountered in her preparation for online teaching was uploading the teaching materials onto the Chaoxing cloud drive. She mentioned the reason why it was a challenge for her.

Jane: *As Chaoxing only operates on its app of mobile phones instead of a software program on computers, how can I transfer teaching materials from my computer to the app? I was totally clueless and frustrated.*

Later, Jane consulted her colleagues and the staff of Chaoxing and learned how to upload the materials to the Chaoxing cloud drive through “Erya,” an online teaching channel run by the university. The materials then can be inserted into the catalog of chapters, synchronized with Chaoxing app, ready for the students to access and download from their smart phones. In the meantime, Jane also learned to develop her courseware on the Chaoxing website and its operations, becoming increasingly familiar with Chaoxing for online teaching.

Similar processes happened to Shirley. She found the difficulties in creating “PPT + voice” files lied in her lively tone as lecturing before her 69 students and the prediction of students’ obstacles in learning.

#### Integrating Traditional Classroom Teaching Into Online EFL Teaching With ICT

In traditional classrooms, before getting into the lessons, Jane would call the roll to make sure all the students were present and ready to learn. She integrated roll calling into her online class as well. The first activity in her online teaching before actual teaching began was roll calling. The roll calling function in Chaoxing often collapsed in the first few weeks as millions of students across China were logging onto the platform for online learning.

Jane: *My targeted countermeasures include extending the roll calling time by starting it half an hour earlier, transferring the roll calling to social networking platforms such as QQ or WeChat groups, or replacing it with random mass calling during lessons.*

Jane found that these countermeasures worked well. They helped ensure the on-time attendance and full participation of her students in her online lessons.

The most important content of teaching in either traditional classrooms or online teaching is to impart knowledge to students and inspire students to think. Some hours before online class time, Jane uploaded the recorded “PPT + voice” video files and PDF teaching materials through the Erya channel into her Chaoxing cloud drive and course folders. In this way the students could access these materials through Chaoxing app on their smart phones. In the online class, Jane asked students to watch the video files and learn the texts with the help of the teaching materials. After watching the videos and self-study, students, started the discussion during which Jane raised questions to provoke students’ critical thinking and analysis, summarize important grammar items in the text, and reflect on their learning processes.

Accurate and effective teaching of EFL is very important in classroom teaching. So Jane integrated this belief into her online teaching by formatting the two 50-min lessons into three sections. The first section started with daily greetings and accurate descriptions about the learning objectives and corresponding learning tasks. Her students were asked to type 1 if they were clear about the tasks. Then the students were asked to spend 20 min or so watching the video files and learning autonomously. They typed 2 when they completed the task. In the second section, Jane raised questions about the knowledge students learned from the videos and the PDF files as described above. The third section began 10 min before the class was over, during which Jane highlighted the key and difficult points and assigned after-class homework for review of what was learned and preview of the next lesson content.

One of the key teaching methods in the traditional classroom teaching process is raising open-ended questions and providing feedback. Open-ended questions can better trigger students’ divergent thinking. Jane applied this method to her own online teaching. She used Text A in Unit One, *Thinking as a Hobby*, as an example to illustrate how she carried this out in her online teaching.

Jane: *In this text, the author explained his own thoughts on thinking and the dynamic development process by describing views and expressions of many characters on thinking in his process of growing up.*

**FIGURE 5:**
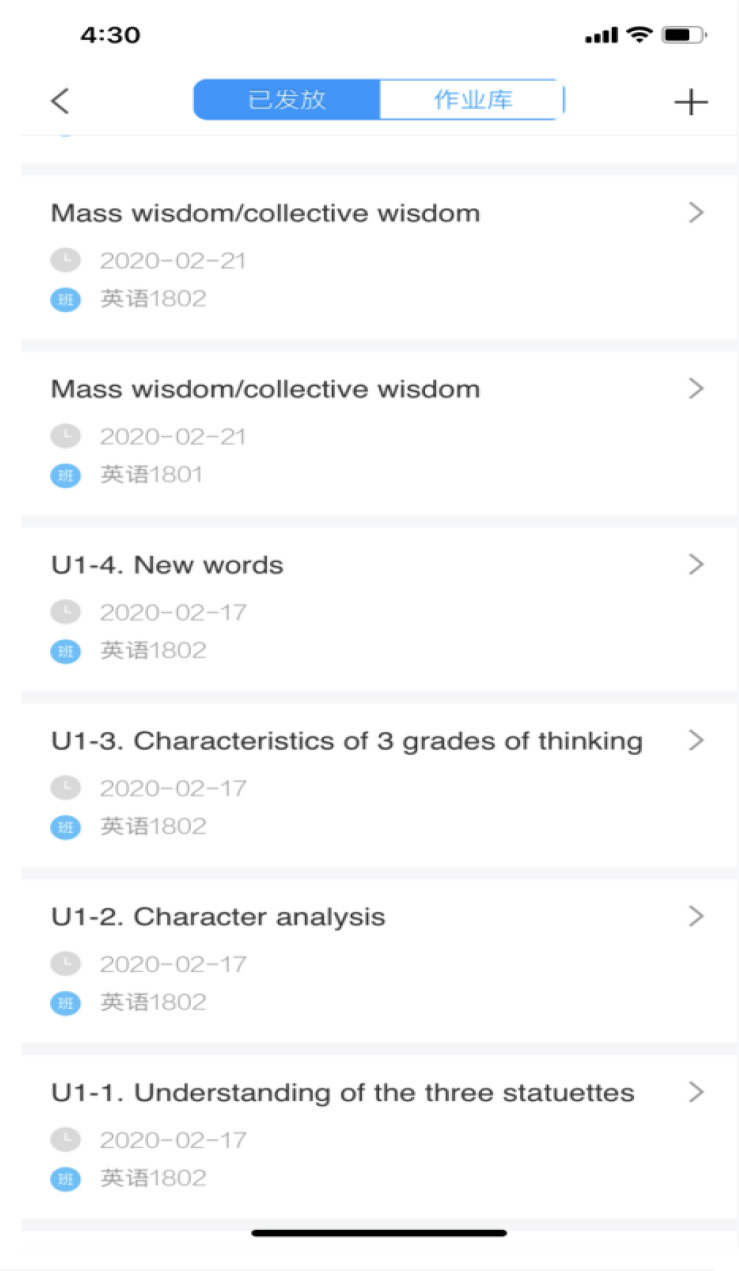
The homework designed by Jane in Chaoxing.

To help students grasp how the author formed and developed his thinking, Jane assigned homework of character analysis through Chaoxing, as shown in Figure 5. The students first listed what the characters said and did as narrated in the text, and then summarized their characters and personalities. In the discussion section of online teaching, Jane led the students to further analyze the features of the characters (such as Mr. Houghton and Ruth) and their thinking. She also asked students find out the role they played in the development of the author’s thinking. In the text, three grading scales for measuring thinking are used (Grade 3, Grade 2, and Grade 1). Grade-one thinking is ranked the highest and indicates the best level of thinking; the characteristics of each grade of thinkers are also illustrated in the text. Through comparison and analysis, the students learned that they should be neither grade-three thinkers full of ignorance and hypocrisy, nor grade-two thinkers who destroy without the power to create; instead they should be trying to be grade-one thinkers who are independent thinkers and set out to seek truth based on logical reasoning.

In view of the research questions stated above, we can see that teachers have identifiable cognitions about online EFL teaching over COVID-19. The interview data and the reflections showed that the participating teachers realized that online EFL teaching, different from traditional classroom teaching, is limited by teachers’ mastery of information technology; online EFL teaching has its apparent advantages such as quality online resources, retrievable online records of students’ learning process and outcome, and lively teacher–student interaction, etc.; meanwhile, online EFL teaching has insuperable deficiencies in its failure to offer instantaneous teacher–student interaction, to monitor their in-class performance, and to ensure education equality caused by imbalance in network devices across regions. This is consistent with Borg’s (2015) model of language teacher cognition: Classroom practice as a contextual factor affects teachers’ cognitions unconsciously and through conscious reflection.

Teachers’ cognitions about online EFL teaching are congruent with their online instructional practice. They chose appropriate platforms and adopted various teaching methods based on their cognitions about students’ learning needs in their courses. This finding is in keeping with the findings of many studies on cognition-practice relationship of language teachers (e.g., Borg, 2011; Basturkmen, 2012; Farrell and Ives, 2015; Li, 2020).

Concerning our second research question, teachers acquired their ICT literacy through their clear understanding of students’ learning needs and was facilitated by online teaching practice and integrating traditional classroom teaching. This finding aligns with the TPACK model developed by Koehler and Mishra (2005) in that TPK is the knowledge domain that has overlaps with TK and PK. Teachers’ integration of their teaching methods in traditional classrooms into online EFL teaching is a new and meaningful finding that adds new empirical evidence to the existing research on teacher cognitions and TPACK.

## Conclusion

This qualitative inquiry, as part of a larger study, was set up to examine EFL teachers’ cognitions about online teaching in response to their disrupted teaching plans, and how they acquired their information technology literacy in the initial stage of the COVID-19 outbreak. Such research on teacher cognition about online teaching during COVID-19 and the findings have theoretical implications for research on online EFL teaching and on teacher cognition. It has modestly expanded the knowledge about online EFL teaching by focusing on what teachers think and believe about online EFL teaching. The qualitative research adopted in the present study has counterbalanced the dominant trend in foreign language research that is predominantly quantitative in methodology. This study has also provided new empirical evidence for research on teacher cognition about online EFL teaching because the findings related to teachers’ cognitions about the features, advantages, and weaknesses of online EFL teaching are new to the field of language teacher cognition research. The findings from the present study might also have pedagogical implications for EFL teachers. These are contextually relevant and meaningful findings in this trying time. These findings further illustrate that research on language teacher cognition needs to be further investigated with reference to specific contexts. Hopefully, our findings can help EFL teachers in the Chinese context or any other similar context in other parts of the world to understand that language teaching is an endeavor full of complexities and unexpected events. Language teachers, as colleagues in other disciplines, need to be flexible, resilient, and ready to learn new skills for tiding over unexpected challenges such as COVID-19.

Despite its significance, our study has one obvious limitation: The lack of data from students. COVID-19 imposed challenges to both teachers and students. Data from students’ perspectives would allow for a more holistic study. Future research might consider including data from students’ perspectives for a more generalizable and holistic study. Teachers’ personal experiences such as birthplaces, schooling, and professional coursework may lead to differences in their cognitions about online EFL teaching. Meanwhile, students’ cognitions about online EFL teaching and learning collected through interviews and/or other data collection instruments would provide feedback to teachers and facilitate their cognitions about EFL teaching, especially online teaching of EFL to students in similar contexts.

## Data Availability Statement

The raw data supporting the conclusions of this article will be made available by the authors, without undue reservation.

## Ethics Statement

Ethical review and approval was not required for the study on human participants in accordance with the local legislation and institutional requirements. The patients/participants provided their written informed consent to participate in this study. Written informed consent was obtained from the participants for the publication of any potentially identifiable images or data included in this article.

## Author Contributions

LG and LZ conceptualized the study. LG collected the data, analyzed them, and wrote the first draft. LZ contributed to the rewriting and revising of the first draft and subsequent revisions. Both authors contributed to the article and approved the submitted version.

## Conflict of Interest

The authors declare that the research was conducted in the absence of any commercial or financial relationships that could be construed as a potential conflict of interest.

## Footnotes

1 https://google.org/crisisresponse/covid19-map
